# Synthesis, Purification, and Characterization of Molten Salt Fuel for the SALIENT-03 Irradiation Experiment

**DOI:** 10.3390/ma17246215

**Published:** 2024-12-19

**Authors:** Pavel Souček, Ondřej Beneš, Pieter Ralph Hania, Konstantin Georg Kottrup, Elio D’Agata, Alcide Rodrigues, Helena Johanna Uitslag-Doolaard, Rudy J. M. Konings

**Affiliations:** 1 European Commission, Joint Research Centre (JRC), 76125 Karlsruhe, Germany; 2 Nuclear Research and Consultancy Group (NRG), 1755 LE Petten, The Netherlands; hania@nrg.eu (P.R.H.);; 3 European Commission, Joint Research Centre (JRC), 1755 LE Petten, The Netherlands

**Keywords:** molten salt reactor fuel synthesis, actinide fluorides synthesis, molten salt fuel irradiation, SALIENT-03 experiment

## Abstract

This work presents the synthesis, purification, and characterization of a molten salt fuel for the irradiation experiment SALIENT-03 (SALt Irradiation ExperimeNT), a collaborative effort between the Nuclear Research and Consultancy Group and the Joint Research Centre, European Commission. The primary objective of the project is to investigate the corrosion behavior of selected Ni-alloy based structural materials which are being considered for the construction of fluoride molten salt reactors. During the test, these materials will be exposed to selected liquid molten fuel salts under irradiation in the High Flux Reactor in Petten, the Netherlands. In addition, the properties and distribution of the fission products formed in the fuel salt during burn-up will be studied by various post irradiation examinations. In the SALIENT-03 fuel, U and Pu fluorides, as fissile material, are dissolved in a carrier melt based on a 78^7^LiF-22ThF_4_ eutectic mixture to form fuel salts with four different compositions, containing PuF_3_, UF_4_, UF_3_, and CrF_3_. This article comprehensively describes all the steps of this fuel synthesis process: the synthesis of the required pure fluoride powders (^7^LiF, ThF_4_, UF_4_, UF_3_, and PuF_3_); the mixing, melting, and purification of the different fuel salt compositions; and the fabrication of solid ingots to be loaded into the irradiation capsules. The characterization of the intermediate and final products is also carried out, following a rigorous quality assurance protocol. The quality assurance is achieved using an analytical scheme consisting of mass balance-based conversion efficiency evaluation, X-ray diffraction, and differential scanning calorimetry analyses. All experimental goals were successfully achieved, highlighting promising prospects for advancing future research and development regarding fuel production methods for fluoride-based molten salt reactors.

## 1. Introduction

### 1.1. Molten Salt Reactor for the Sustainable Energy Production

Nuclear energy has a large potential to contribute significantly to reducing the CO_2_ emissions that are at the source of worldwide climate change. In 2021, the production and use of electric energy generated more than 75% of the greenhouse gas emissions in the European Union [1]. Decarbonizing the EU’s energy system is therefore critical to reaching the climate objectives and carrying out the EU’s long-term strategy of achieving carbon neutrality by 2050. According to the United Nations’ Intergovernmental Panel on Climate Change (IPCC) evaluation, the median value of the life cycle CO_2_ equivalent of nuclear energy is 12 gCO_2_eq/kWh. This is the same as that of offshore wind energy, half that of hydroelectric power generation (24 gCO_2_eq/kWh), 25% of the value of solar power (48 gCO_2_eq/kWh), and only 2.4% and 1.5% of the values for electric power production using gas and coal (490 and 820 gCO_2_eq/kWh, respectively) [2,3].

To achieve safe, sustainable, and economical nuclear energy production, new reactor concepts are being developed worldwide. The Molten Salt Reactor (MSR) is one of the advanced reactor concepts that has been studied within the Generation IV Forum since 2002 [4,5,6]. The main advantages of this type of reactor are its inherent safety due to its strong negative temperature coefficient of reactivity and improved sustainability. The MSR is operated at atmospheric pressure in the primary circuit, and in the event of overheating, the fuel would be drained into an emergency dump tank, assuring subcriticality and natural removal of the decay heat. More details on these and other MSR safety performance features are given in [7]. The interest in MSR technologies is increasing worldwide, and not only within national programs, e.g., in France [8], the USA [9], China [10], and others, as a number of concepts are being developed commercially by several start-up companies, e.g., Naarea, Stellaria (France), Seaborg Technologies, Copenhagen Atomics (Denmark), Thorizon (Netherlands), Terrestrial Energy (Canada/USA), TerraPower (USA), Moltex (UK), and others.

### 1.2. Irradiation Experiments of Molten Salt Reactor Fuel Candidates

In many of the proposed MSR designs, molten salt is used as a carrier for the dissolved fuel compounds, while serving at the same time as a primary coolant of the reactor and, in the case of fluoride salt-based MSRs, as neutron moderator. Despite the significant developments made in different MSR systems, the data on the behavior of molten salt fuels under irradiation are still lacking. To address this point, the Nuclear Research and Consultancy Group (NRG, Netherlands) and the Joint Research Centre (JRC, European Commission) have been collaborating since 2015 on a study of the in-pile and post-irradiation behaviour of liquid molten salts, which are being considered as possible candidates for MSR fuel, under the program LUMOS (Learning to Understand MOlten Salts).

The first irradiation of liquid molten salt composed of 78^7^LiF-22ThF4 was carried out within the experiment SALIENT-01 (SALt Irradiation ExperimeNT), commenced under the same program as this study. The salt was irradiated in the High Flux Reactor (HFR) in Petten (the Netherlands) for 2 years between 2017 and 2019, and the post-irradiation analyses are ongoing [11]. The main goal of the follow-up experiment SALIENT-03 is to assess the mechanisms and corrosion rates of selected Ni-based alloys in molten fluoride salt during irradiation in the HFR. In addition, the properties and distribution of the fission products formed in the fuel salt during burn-up will be studied by various post-irradiation examinations. The fuel is based on the same 78^7^LiF-22ThF_4_ eutectic mixture as in the previous experiment, SALIENT-01. The significant difference is that, in the SALIENT-03 fuel, U and Pu fluorides, as fissile material, are dissolved in this carrier melt to form a salt with a composition of 75.0^7^LiF-18.7ThF_4_-6.0UF_4_/UF_3_-0.3PuF_3_. All technical details and targets of the SALIENT-03 experiment can be found in [12]. In both SALIENT experiments that have been carried out to date, the selected salt composition represents the carrier salt considered for use in the Molten Salt Fast Reactor (MSFR) concept studied in Europe [8]. The MSFR is based on a non-moderated neutron spectrum utilizing the ^232^Th-^233^U fuel cycle. It was developed in France and, since 2015, its safety assessment is being conducted by the R&D projects in the frame of the EC/EURATOM programs [13,14,15].

### 1.3. Preparation of the Fuel for the SALIENT-03 Experiment

The present article is focused on the synthesis of the fuel for the SALIENT-03 experiment, describing all the steps of the process: (i) the synthesis of the required fuel end-members; (ii) the mixing, melting, and purification of four different fuel salt compositions; and (iii) the fabrication of solid fuel ingots to be loaded into the irradiation capsules.

#### 1.3.1. Synthesis of the Pure Fluorides for the SALIENT-03 Fuel

The required end members were ^7^LiF, ThF_4_, UF_4_, UF_3_, and PuF_3_. Lithium fluoride is commonly prepared through the reaction of lithium hydroxide or lithium carbonate with hydrogen fluoride [16,17]. However, alternative methods are reported in the literature, such as using silicofluorides to precipitate LiF from a LiOH solution [18] or reacting lithium chloride with ammonium fluoride [19]. Since the SALIENT-03 fuel salt required lithium enrichment to 99.9% ^7^Li, and the only available source material of enriched lithium was ^7^LiOH·H₂O, it was decided to use a method based on precipitating LiF from the hydroxide solution using hydrogen fluoride. The synthesis of UF₄, ThF₄, and PuF_3_ followed a procedure previously developed by the JRC. Detailed information on the applied procedure, along with an extensive review of alternative methods, can be found in references [20,21]. On the other hand, a new method for the synthesis of UF_3_ was developed, as described in Section 3.1 below. This method is based on the reduction of UF_4_ by H_2_ gas as reported in [22,23], but was adapted to use a reaction gas consisting of H_2_ diluted to 6% with Ar in order to meet the safety requirements of the available experimental setup. Only a few alternative methods were found, e.g., Runnalls achieved the reduction of UF_4_ to UF_3_ by using pure Al [24] and Gilpatrick produced UF_3_ through the reduction of UF_4_ with U metal [25].

#### 1.3.2. Preparation of the Fuel Salts from the End Members

With the growing global interest in MSR technology, MSR fuel preparation and the chemistry involved in the process have been discussed in many recent publications. These include, e.g., the joint NEA-IAEA Workshop on the Chemistry of Fuel Cycles for Molten Salt Reactor Technologies [26], contributions to the US Nuclear Regulatory Commission’s Regulatory Information Conference W19 on Molten Salt Reactors: Rethinking the Fuel Cycle [27,28], and the Molten Salt Reactor Fuel Cycle Chemistry Workshop organized by the US Department of Energy [29]. Other sources include reports from US National Laboratories on MSR fuel salt processing [30] and qualification [31], as well as recent works from the JRC in this area [32,33,34]. However, the preparation of the fuel for the SALIENT-03 experiment was unique, given the specific experimental, technical, and safety constraints. It did not follow any existing process described in the literature. The procedures for mixing, melting, and purifying the fuel salt mixtures, and for converting them into solid fuel ingots, were designed specifically for this experiment, as described in Section 3.2.

The characterization of the intermediate and final products is mentioned, following the quality assurance (QA) protocol. The QA protocol was validated through the use of an analytical scheme established in JRC laboratories during previous work on the synthesis of actinide fluorides for use as MSR fuel candidates [20,21,32]. The techniques used are described in Section 2.4 below, and include the mass balance of the conversion efficiency, X-ray diffraction (XRD), and differential scanning calorimetry (DSC) analyses.

## 2. Experimental

### 2.1. Fuel Salts Specification

The fuel for the SALIENT-03 experiment consisted of four salt mixtures with different compositions, defined by the irradiation experiment design developed by NRG and summarized in Table 1, including the acronyms for the mixtures used throughout the text (Fuel-1 to Fuel-4) and the masses required for their synthesis. The masses were calculated from the volumes required for each of the irradiation capsules described in Section 2.2 and the estimated density of the salt (2.7 g/cm^3^). Three fuel mixtures (Fuel-1 to Fuel-3) are planned to be used for the irradiation and one for an out-of-pile electrochemical experiment (Fuel-4).

Table 2 summarizes the end-members needed and their masses to be synthesized, calculated from the required composition with and added 20% of extra material allocated for sampling for the QA protocol and expected losses during the overall process. Fuel-4 is specified in more detail in Section 3.3, as its synthesis is described separately.

### 2.2. Irradiation Capsules

The fuel salts intended for the irradiation will be filled into 6 capsules (1–5 and D1) designed and manufactured by NRG using the materials selected for the corrosion test, i.e., Hastelloy N (1–4 and D1, Haynes International, Inc., Kokomo, IN, USA) and GH3535 (5, SINAP, Shanghai, China). Hastelloy N is a nickel–molybdenum–chromium alloy developed by Oak Ridge National Laboratory (US), initially under the name INOR-8, and specifically tailored to withstand the corrosiveness of the fluoride-based MSR fuels. GH3535, developed by the Shanghai Institute of Applied Physics (SINAP), Chinese Academy of Sciences, has an almost identical chemical composition. Table 3 shows the distribution of the fuel mixtures in the irradiation pins and their required masses.

**Figure 1:**
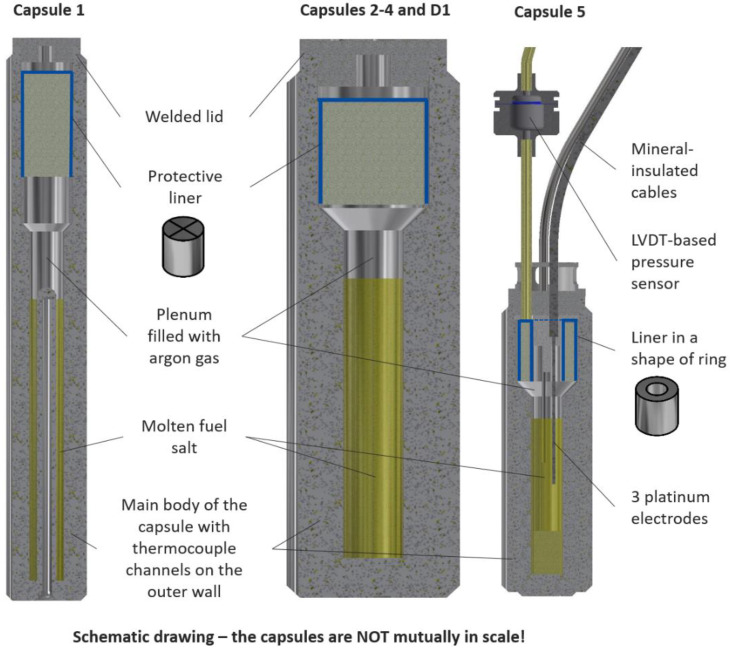
Schematic drawing of the SALIENT-03 irradiation capsules.

The SALIENT-03 irradiation sample holder contains 5 of the fuel capsules, while the D1 capsule is intended for a reference out-of-pile corrosion test under the same thermal conditions to facilitate an evaluation of the effect of the irradiation. Each pin holds a certain volume of the fuel salt and argon cover gas. The capsules come in three basic geometries as illustrated in Figure 1 by schematic cross-sections of the capsules. They are thick-walled cylinders with an inner diameter of 7 mm, and include thermocouple channels along the outer wall and a welded lid. Capsule 5 connects to a linear variable differential transformer-based pressure sensor (IFE, Halden, Norway) and includes 3 platinum electrodes introduced as mineral-insulated cables (Thermocoax, Caligny, France), which will be connected to a potentiostat. Capsule 1 is longer, contains twice the amount of salt, and has an annular salt volume with a provision for 3 central thermocouples in a central insert. A detailed specification of the capsules and the whole irradiation assembly is given in [12].

After the fuel salt is introduced, the pins are closed using a lid welded to the upper part by orbital welding. Since the welding facility is installed in a contamination-free glove box at JRC Karlsruhe, the surfaces of the capsules with the fuel have to be free from any radioactive contamination before the welding. This implies that the fuel salt cannot be inserted in the form of powder, and it was decided to introduce it to the pins in the form of solid ingots, which enabled easy surface decontamination of the pins. In addition, the capsule is in a horizontal position during the welding, which brings additional risk of re-melting of the fuel salt ingot due to transfer of the heat and a possible leakage of the liquid melt to the welded zone during the welding. To reduce this risk, a liner is placed in the upper part of the plenum of each capsule. The liners are made of the same material as the pins in which they are inserted. In the case of pins 1–4 and D1, the liners have a cylindrical shape, while pin 5 has a ring shape with a central hole in the center to allow the electrodes to come through, as shown in Figure 1.

### 2.3. Experimental Set-Up for the Synthesis

The synthesis of the actinide-containing end members was carried out in a facility installed at JRC Karlsruhe, which is exclusively designed for the fluorination of radioactive actinide materials using pure HF gas. The main parts of the set-up consist of a high-temperature horizontal fluorination reactor in the glove box and a connected HF supply gas line. A detailed description of the complete installation is given in [20]. The fluorination reactor was also used for the melting of the salt mixtures to prepare and purify the fuel ingots. The chemicals used and particular details of the set-up are specified within the individual sections describing each step of the fuel preparation. Handling and storage of the materials during the fuel preparation was done in a glove box under a purified Ar atmosphere that was controlled to keep the concentration of H_2_O and O_2_ less than 5 ppm (peak value during certain manipulations), while it was typically less than 2 ppm in normal working conditions.

### 2.4. Analytical Scheme

The purity of the fuel was controlled by a combination of different analytical techniques, whose technical specifications are described in [20,21,32]. The synthesized end members were characterized by gravimetric the mass balance efficiency of the reaction, by X-ray diffraction (XRD) for the qualitative phase analysis using Rietveld refinement, which provided crystallographic cell parameters and quantification estimates of the phases with a detection limit typically considered as 1 wt.%, and by differential scanning calorimetry (DSC) for the melting temperature determination, with an uncertainty of ±5 °C. Melting point determination by DSC is renowned as a very sensitive technique for the evaluation of whether some impurities are present in a sample, which in most cases includes oxygen containing compounds, based on the value of the melting point and the shape of the heating curve. The salt mixtures before melting were analyzed by DSC and, in addition, one selected ingot representing the final form of the fuel before its insertion into the capsules was analyzed by XRD. A direct oxygen analysis in the actinide containing halides is technically very demanding. It requires an instrument installed in a radiological glove box, which at the same time is required to be chemically resistant towards very aggressive chemicals due to the possibility of fluorine gas being released during the analysis. Therefore, no equipment is available for the direct quantitative oxygen analysis of the synthesized materials.

## 3. Results

This chapter summarizes the results of the synthesis of all the end members, ^7^LiF, ThF_4_, UF_4_, UF_3_, and PuF_3_. It also outlines the fabrication process of Fuel-1 to Fuel-3, which involved mixing and homogenizing the end members into the required fuels and subsequently preparing the fuel ingots. Furthermore, the fabrication of Fuel-4, which was planned for out-of-pile tests, is described separately. The main process parameters and mass balances are described in the text in a comprehensive manner, with the conditions also being mentioned. These led to unsatisfactory results and had to be optimized. In addition, the procedures used to achieve satisfactory results for all the main steps are presented in tabular form in the Supporting Material: Annex S2, which provides a clear summary of the technical details of the overall SALIENT-03 fuel fabrication process.

### 3.1. Synthesis of the End-Members

The ^7^LiF end member was synthesized from the commercially obtained ^7^LiOH⋅H_2_O (Sigma-Aldrich, Burlington, MA, USA, 99.9% ^7^Li enrichment, MQ level 200) according to the below-listed steps. For all reactions, deionized water of a purity corresponding to Milli-Q grade or better (resistance > 18.2 MΩ⋅cm^−1^, prepared at JRC Karlsruhe) was used to limit contamination from other naturally occurring cations. The ^7^LiF was synthesized according to the following steps:

1. The dissolution of app. 20 g of ^7^LiOH⋅H_2_O in water to a concentration of app. 2 M (concentration is estimated as the initial material contained traces of ^7^Li_2_CO_3_ and moisture);
2. The filtration of the undissolved ^7^Li_2_CO_3_, which was partly formed in the delivered package, likely due to CO_2_ intake during open handling of the material between manufacturing and delivery;
3. The reaction of the ^7^LiOH solution with hydrofluoric acid solution (HF, 49 wt.%, Sigma-Aldrich, Nuremberg, Germany, p.a. plus, diluted to 2 M concentration), controlled online by a commercial pH meter with a calibrated pH electrode (InLab Routine, Mettler Toledo GmbH, Gießen, Germany), until it reached pH ~4–5;
4. The filtration under a vacuum of the formed solid, ^7^LiF;
5. The rinsing of the ^7^LiF with pure ethanol (p.a., Sigma-Aldrich, Nuremberg, Germany);
6. The drying of the product at 150 °C for 2 h in air;
7. The drying of the product at 350 °C under argon in the glove box described above in Section 2.3

The overall reaction efficiency was estimated to be >90%; however, it cannot be exactly calculated as the initial material contained traces of ^7^Li_2_CO_3_ and moisture. XRD of the final product indicated a composition of single-phase LiF; however, likely due to low crystallinity of the material, the background at low 2θ is very high and the results cannot be taken as fully conclusive. The DSC heating curve revealed a single peak, confirming the phase purity, and a melting point of 846.8 °C, which is in excellent agreement with the reference value of 847.8 °C [35]. Although no impurity was identified, the purity was declared as >99.0% due to the detection limit and the uncertainties of the used methods. The XRD and DSC results are shown in Figures S1 and S2, respectively, in the Supporting Material: Annex S1.

The ThF_4_, UF_4_, and PuF_3_ end members were synthesized according to the procedures previously established at JRC Karlsruhe and published in [16,18]. Within the presented campaign, the procedures were proven to be reproducible and usable for significantly larger-scale synthesis than in the published works. In all cases, the method was based on the solid-gas fluorination of stoichiometric high-surface AnO_2_ (An = Th, U, Pu) by pure HF gas at elevated temperatures using a gas flow-through technique. In the case of PuF_3_, the fluorination was followed by reduction of the intermediate product by Ar-H_2_(6%) gas. More details are given in Table 4, summarizing the particular parameters that were used and the mass balances of the syntheses, including the mass of the product after the reaction (*m*_AnFx_) and the mass of the recovered product after sampling (*m*_AnFx_FINAL_). Due to the limited capacity of the fluorination reactor, which was able to hold app. 15 g of the initial material, the ThF_4_ was synthesized in four batches and the UF_4_ in two batches. The flow rate of the HF gas was 50 mL/min for all of the batches; therefore, only the reaction time is presented. The batch numbering (from ThF4-1 to ThF4-4, UF4-1 and UF4-2) only indicates the sequence in which the batches were synthesized and is not related to the numbering of the fuel batches (from Fuel-1 to Fuel-4) presented in Section 2.1.

The initial oxide powders were prepared using the published procedures [16,18], where the main step is calcination of the respective oxalates at 800 °C (Th, U) and 600 °C (Pu). The source materials’ U(VI), Th(IV), and Pu(IV) nitrate solutions were stock material of JRC Karlsruhe for which the isotopic compositions are published in [12].

In all cases, the products were homogeneous powders with the expected appearance, i.e., white for ThF_4_, dark green for UF_4_, and violet-purple for PuF_3_. All XRD patterns revealed only the peaks corresponding to the pure compounds, indicating phase purity. The DSC heating curves all yielded a single peak with no shoulders, which confirmed the phase purities, and the derived melting points were in good agreement with the published values for ThF_4_ [20,36] and UF_4_ [20,37]. Since the melting point of PuF_3_ is higher than that of the available encapsulating material used for the DSC measurement, this analysis could not be done. Although no impurities were identified, the purity of each compound was declared as >99.0% due to the detection limit and uncertainties of the used methods. The selected typical XRD and DSC results for the synthesized ThF_4_, UF_4_, and PuF_3_ are shown in Figures S3–S7 in the Supporting Material: Annex S1.

The method for UF_3_ synthesis established at JRC Karlsruhe and applied in this synthesis campaign has not yet been published and, therefore, it is described in more detail in this separate section. The procedure is based on the solid-gas reduction of UF_4_ by H_2_(6%)/Ar gas at an elevated temperature according to the equilibrium (1):

2UF_4_(s) + H_2_(g) ↔ 2UF_3_(s) + 2HF(g). (1)

The H_2_ gas had to be diluted in view of the safety restrictions applied in the laboratory, which do not allow the usage of pure H_2_. During the previous yet unpublished experimental work on the synthesis of UF_3_ conducted at JRC, the minimum efficient temperature for the reduction with the diluted H_2_ was determined to be 800 °C. In the same amount of time, when using a typical flow rate of the reaction gas of about 50–100 mL/min, the UF_3_ product was already found to be partly disproportionate to the UF_4_ and U metal at this temperature. It was empirically shown that, to suppress the disproportionation reaction and to overcome the unfavorable kinetics, the reaction has to be carried out with a high flow-rate and using a large excess of H_2_. This fact brings the risk of an unwanted side reaction of the initial UF_4_ or of the product having traces of the oxygen that is usually present in the H_2_(6%)/Ar reaction gas, forming a measurable amount of UO_2_ in the product.

**Figure 2:**
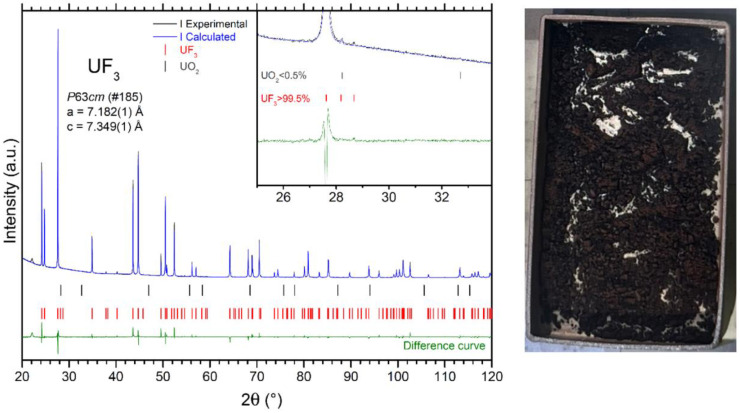
XRD result of the UF_3_ synthesis showing a phase purity >99.5% for UF_3_ and <0.5% for UO_2_ (**left**), and photo of the final UF_3_ product (**right**). The crystallographic cell parameters for UF_3_ phase determined from the Rietveld refinement are shown in the graph.

In this work, about 1 g of the UF_4_ powder synthesized during the previous step was introduced, in a nickel boat (99.95%), into the fluorination reactor, which was then closed, evacuated, filled with pure Ar gas, and heated to 800 °C. Then, H_2_(6%)/Ar gas was introduced into the reactor at a flow-rate of 600 mL/min for 40 h. The product was homogeneous powder with a very dark violet, almost black, color, as illustrated in Figure 2. The process parameters, mass balance, and results of the UF_3_ synthesis are summarized in Table 5. The XRD analysis shown in Figure 2 indicated the possible presence of UO_2_, although in a very low concentration which was estimated by Rietveld refinement and quantification to be less than 0.5 wt.%. The results cannot be taken as fully conclusive, as only one diffraction peak, the most intensive diffraction peak from the UO_2_ spectrum, was detected at 28.2° 2θ; in addition, UF_3_ has a weak diffraction peak in the same region, as shown in the inset of Figure 2. The DSC analysis could not be conducted due to disproportionation of the UF_3_ before it reached its melting temperature. Therefore, since the typical detection limit of the XRD technique is considered to be 1 wt.%, the purity of the synthesized UF_3_ was declared in the same way as the other compounds to be >99.0%.

### 3.2. Fabrication of Fuels-1, -2 and -3

All Fuel-1 to Fuel-4, were mixed in glass mixing bottles through step-by-step additions of the required end-members to form compositions as specified above in Table 1. The commercially available CrF_3_ (Merck, anhydrous, 99.99%, Darmstadt, Germany) was purified by drying under argon gas and by fluorination in a stream of pure HF gas at 400 °C for 2 h. Due to the fact that only a trace amount of CrF_3_ was added to Fuel-3, the analyses of the purified material were omitted. The salts were homogenized by, first, mixing for 10 min with a spoon inside the bottles, then by shaking the bottles for 30 min, and, finally, by thorough grinding in a large agate mortar in multiple batches. The masses of each end-member used, the total mass (*m*_FUEL_), and the final mass after homogenization (*m*_FINAL_) are given in Table 6. Fuel-1 to Fuel-3, intended for irradiation, were examined by DSC measurements, which showed the same melting behavior and similar melting points of all three mixtures. No indication of liquidus-type equilibria was observed in both the heating and cooling curves, indicating that the compositions corresponded closely to the eutectic point, which was expected due to them having comparable compositions to the major content of eutectic LiF-ThF_4_ mixture. The measured melting points were 547.6, 549.2, and 543.6 °C for Fuel-1, Fuel-2 and Fuel-3, respectively. The details of the DSC heating curves for Fuel-1 to Fuel-3 are summarized in Figures S8–S10 in the Supporting Material: Annex S1.

As detailed in Section 2.2, the fuel salts cannot be inserted into the irradiation pins as powders, and must be inserted in the form of solid ingots fitted either directly to the capsules or to the liners placed inside the capsules. The ingots for each pin were prepared by multiple melting and cooling cycles of the fuel salt in a suitable mold, according to the procedure described below. The sub-ingots are named using the number of the capsule for which the sub-ingots were used (1–5 and D) and an identification number of each sub-ingot. E.g., sub-ingot 1-3 refers to sub-ingot number 3 inserted to capsule 1.

The ingots for capsule 1-4 and D1 were prepared using the corresponding liners as molds. However, in order to fulfil the required fuel salt mass in each capsule, the ingots were required to have almost exactly the same volume as the liner. Therefore, for each capsule, four sub-ingots had to be prepared and fused together to form the final ingots at the last stage of the fabrication process. The reason for this is significant shrinking of the salt volume during the initial melting step corresponding to the transition from mixed powders to eutectic mixture: the volume of the solidified salt after melting decreased to only 25% of the volume of the compressed powder. The final fuel salt ingots were then placed into the capsules before the welding of the lids inside the liners.

In the case of capsule 5, the volume of the ring-shaped liner was not enough to contain all the salt. It was filled as much as possible and the remaining required amount of fuel salt was introduced in the form of two additional ingots placed directly inside the main body of the capsule. Since the diameter of the main body of capsule 5 is smaller than the diameter of the cylindrical liners, these additional ingots were fabricated from four thin sub-ingots prepared in glassy carbon crucibles, which had exactly the required diameter. In addition, two extra small sub-ingots were prepared for the QA check of the final ingots. These sub-ingots are referred to as GC-1–GC-6.

**Figure 3:**
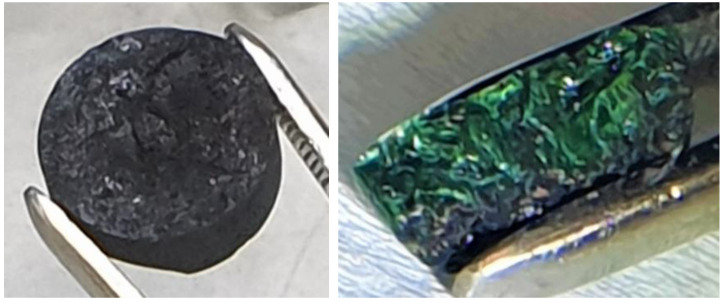
Fuel salt sub-ingot prepared under argon atmosphere—surface (**left**) and cross-section (**right**).

The initially attempted procedure for the sub-ingots’ fabrication was based on melting of the fuel salt powders inside the liners under an argon atmosphere in the glove box, but it did not bring satisfactory results. The fuel salts were filled into the liners, mechanically compressed, and introduced into a furnace placed in the same glove box that was used for the synthesis. The salts were melted in the liners at a temperature of 700 °C for 30 min and, after cooling to room temperature, the formed sub-ingots were removed from the liners. The sub-ingots were covered with a black layer, which could not be mechanically removed (see Figure 3 left). The XRD analysis of the layers from two selected sub-ingots revealed 6 and 10 wt.% of UO_2_. The cross-section of the sub-ingots showed visually homogeneous green bulk and only a thin black layer on the surfaces, as illustrated in Figure 3 on the right. Although this indicated that the impurity layer represented only a minor part of the sub-ingot, it was not acceptable as the final product, so detailed analyses were avoided due to time constraints and a new procedure for preparation of the sub-ingots was developed. The origin of the oxygen remained unclear; however, traces of oxygen present in the Ar gas were assumed to be the main possible source. Oxygen from oxides possibly present at the liner walls was not considered as an important source, as the liners were treated beforehand in a reducing atmosphere of H_2_/Ar(6%H_2_) gas at 800 °C for 1 h, which should minimize the oxygen impurities on their surfaces.

The advanced procedure consisted of melting the fuel salts in the liners under a flow of pure HF gas instead of Ar. The liners were filled with the fuel salt powder and introduced into the same fluorination reactor that was used for the syntheses. The reactor was evacuated to an absolute pressure of 1 mbar and filled with pure HF gas, while having already been heated during the filling to a temperature of 700 °C in 1 h ramp-ups. When the absolute pressure of HF in the reactor reached 1 bar, HF was introduced into the reactor with a flow rate of 50 mL/min. The flow was launched at a temperature inside the reactor of about 200 °C, i.e., before the salts were molten. The salts were then melted for 30 min under the HF gas flow at 700 °C, followed by cooling the reactor down as quickly as possible, still under the flow of HF gas, reduced to 25 mL/min. When a temperature of about 300 °C was reached in the reactor, the HF flow was stopped and the reactor was isolated under an HF atmosphere. At a temperature of 100 °C, the reactor was flushed with argon gas and allowed to cool down overnight under the argon flow to remove all HF from the system. The additional sub-ingots, GC1–6 for pin 5, and the QA protocol were prepared using the same procedure but in glassy carbon crucibles with an inner diameter of 6 mm and height of 10 mm.

**Figure 4:**
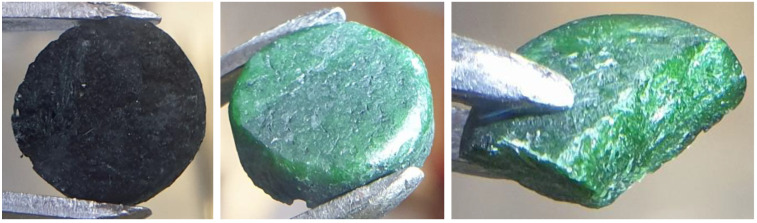
Fuel salt sub-ingot D1-1 prepared under HF atmosphere—surface before cleaning (**left**), surface after cleaning (**middle**), and cross-section after cleaning (**right**).

The obtained sub-ingots were weighed in the liners, removed from the liners, mechanically cleaned by a brush, shortly rinsed in ethanol, and dried and weighed again. The sub-ingots prepared in this advanced way still had a thin dark surface layer, but it was possible to remove the layer mechanically, yielding pure sub-ingots that were homogenously green both on their surface and in their bulk, as shown in Figure 4 in the example of ingot D1-1. The liners were cleaned by ethanol and weighed to detect possible corrosion during the melting.

The preparation of all sub-ingots brought very similar results, i.e., comparable mass balances and melting behavior. A list of all fabricated sub-ingots is presented in Table 7 together with the most important mass balance data, while the complete mass balances of the sub-ingots for capsule 1 are shown in Table 8 as a representative example. The mass balance includes the mass of the powder before melting (*m*_SALT_), the mass of the sub-ingot after melting (*m*_INGOT_), the corresponding mass loss (Δ*m*_MELTING_), and the estimation of the maximum mass of the black layer (*m*_BLACK_L_) done by weighing the sub-ingots before and after cleaning. The exact content of the black layer cannot be determined as the mass difference possibly also includes some minor losses from the clean sub-ingot during manipulation and cleaning. The average mass difference between before and after the cleaning, taken from all prepared sub-ingots, was 0.23 wt.%. In addition, Table 8 shows the mass differences of the liners between before and after melting (Δ*m*_LINER_), indicating negligible corrosion, and photos of the sub-ingots.

**Figure 5:**
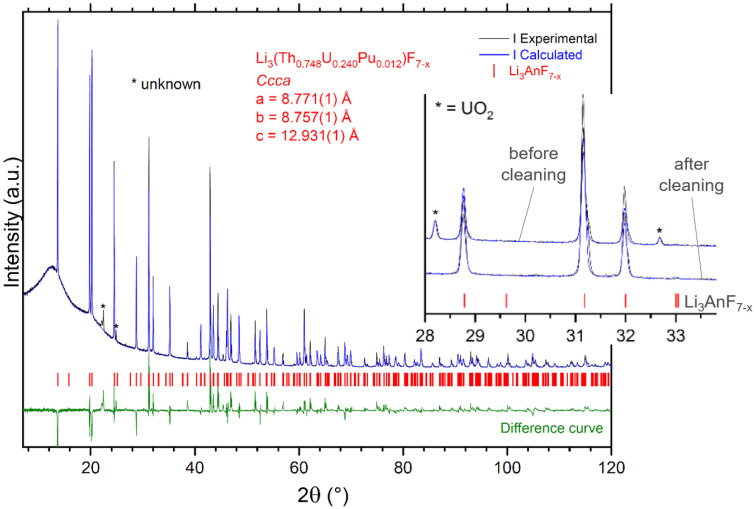
XRD pattern of the clean sub-ingot (**left**) and a zoomed-in comparison of the XRD patterns before and after cleaning of the black layer, focusing on a key region (**right**). The crystallographic cell parameters for the single detected phase, Li_3_(Th_0.748_U_0.240_Pu_0.012_)F_7−x_, determined from the Rietveld refinement, are shown in the graph.

XRD analysis of sub-ingot GC5, prepared for the QA of the final ingots (Fuel-2), revealed a composition of phase pure Li_3_(Th_0.748_U_0.240_Pu_0.012_)F_7−x_ without any traces of UO_2_, as shown in Figure 5 (left). Detail of the XRD patterns corresponding to the cleaned sub-ingot and to the black layer from the sub-ingots prepared under an argon atmosphere are shown in Figure 5 (right), which clearly shows the presence of UO_2_ in the black layer but not in the cleaned ingot.

**Figure 6:**
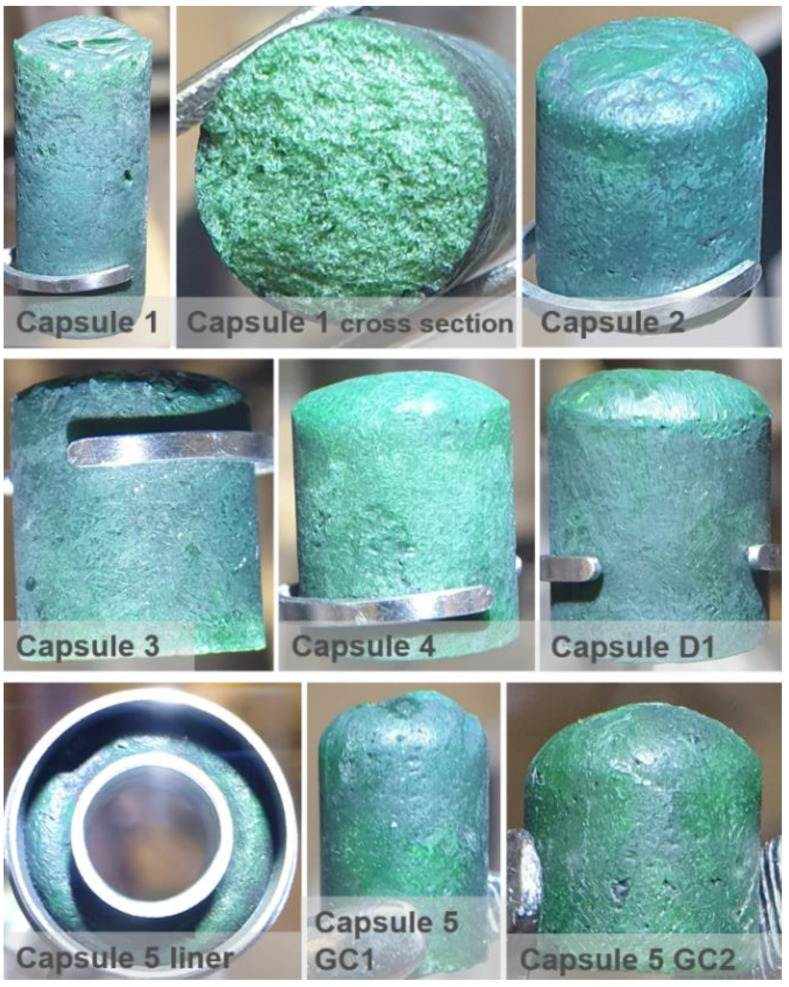
Photos of the final ingots.

The final ingots were prepared by fusing together the respective sub-ingots by melting them under a pure HF gas atmosphere. Although all the sub-ingots were cleaned, a very thin black layer was again formed on the surface of the final ingots. However, it was possible to very easily mechanically remove the layer, as it was not prominently adherent and the estimated mass of the layer was lower than in the case of preparation of the sub-ingots, on average being 0.10% of the total ingot mass. The basic mass balances of the ingots prepared using the final preparation methods are summarized in Table 9, together with the estimated densities of the ingots and a comparison of the achieved ingot mass for each irradiation pin with the requested mass (Δ*m*_REQUEST_). The density of the solid ingots was roughly estimated from their dimensions and masses, disregarding minor shape irregularities. The obtained values are consistent with an average of 4.5 g/cm^3^, which corresponds well to the estimated value of 4.3 g/cm^3^ of the liquid fuel salt at 727 °C. This also indicates that the ingots are likely compact without major cavities, which was confirmed by a random cross-section check of the final ingot for capsule 1, shown in Figure 6 together with photos of all final ingots.

### 3.3. Fabrication of Fuel-4

The required composition of Fuel-4 was 75LiF-23.0ThF_4_-2.0UF_4_-0.1UF_3_ (mol. %) with an initial UF_4_/UF_3_ ratio 100:1, while additional UF_3_ was to be delivered separately and added to the melt during the experimental campaign to increase the UF_4_/UF_3_ ratio up to 20:1. The required total mass was 50.0 g. The LiF was purchased from Alfa-Aesar (99.99 wt.% anhydrous, packed under Ar), and it was used as received and purified later in the final mixture by HF gas bubbling. The ThF_4_, UF_4_, and UF_3_ material was synthesized together for all Fuel-1 to Fuel-4, as described above in Section 3.

**Figure 7:**
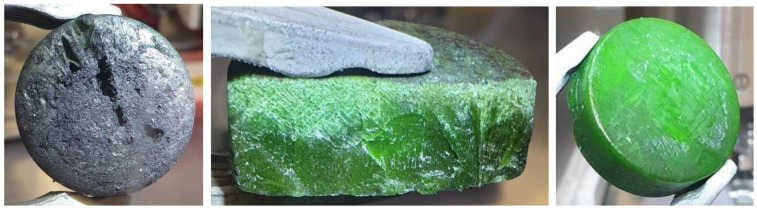
Photos of Fuel-4 after melting under Ar atmosphere (**left**—surface, **middle**—cross-section) and after purification by fluorination (**right**).

Fuel-4 was mixed and homogenized using the same procedure as described above in Section 3.2 for Fuel-1 to Fuel-3. To obtain a form suitable for transport, it was melted in a glassy carbon crucible under an argon gas atmosphere in the glove box, i.e., H_2_O and O_2_ < 5 ppm, at a temperature of 700 °C for 1 h. The crucible was previously thermally treated by heating to 800 °C for 8 h under the same atmosphere. The resulting salt puck weighed 54.8673 g, losing 0.0775 g (0.14 wt.%) from the original 54.9448 g, likely due to evaporation during the melting. As shown in Figure 7, the upper surface of the salt was covered by a very thin black layer, which could not be completely mechanically removed. The side and bottom surfaces were not fully covered, but had irregular black spots. The ingot was broken in half in order to see the bulk, which was clean and homogeneously green, showing that the black layer was very thin and only on the surface. The surface and a cross-section of the salt puck are shown in Figure 7.

To fully eliminate the black layer, the salt was purified by fluorination in a molten state using pure HF gas, bubbled directly into the salt, which was placed into a glassy carbon crucible at a temperature of 750 °C. HF gas was introduced into the melt through a nickel tube introduced inside the melt for 2 h at a flow rate of 6 mL/min, which was previously determined as the maximum possible rate without splashing the salt from the crucible. After the fluorination, pure Ar gas (6.0 purity) was bubbled for 4 h at 700 °C into the melt at the same flow rate to remove the dissolved HF. The resulting salt had a weight of 53.7760 g and it was homogeneously green without any traces of the black layer, as shown in Figure 7.

## 4. Discussion

The outcomes of the fabrication campaign for the molten salt fuel in the SALIENT-03 irradiation experiment can be categorized into three key areas. First, the verification of previously established methods for synthesizing pure actinide fluorides on a 10 g scale. Second, the development of a new method for synthesizing UF_3_. Lastly, gaining experience in the production of solid melt ingots from mixtures of actinide fluorides.

### 4.1. Outcomes from the Synthesis of Actinide Fluoride End-Members

The main outcome from the synthesis of the actinide fluoride end-members is that the method based on the use of heterogeneous solid oxide and gaseous hydrogen fluoride at elevated temperatures, previously established at JRC Karlsruhe for 1 g-scale production, is fully effective for synthesizing much larger batches (~15 g ThF_4_, ~10 g UF_4_, and ~3 g PuF_3_). This method also appears to yield actinide fluorides of higher purity and/or in a significantly simpler way compared to alternative methods that use different fluorination agents and/or initial materials, as discussed below for each synthesized compound separately.

A literature survey on the synthesis of ThF_4_ indicated that the most efficient method is the fluorination of thorium oxide by anhydrous hydrogen fluoride gas at elevated temperatures [38,39,40], which agrees very well with the results of our work. Opposite to the experiences with UF_4_ synthesis that are described below, in order to obtain pure ThF_4_, the reaction temperature had to be higher in our case (650 °C) than the one used in other studies, typically 400–600 °C [40]. However, the high-purity ThF_4_ product was achieved in our work through a simpler process, in a single reaction step, than that of UF_4_ synthesis, which typically requires at least two consecutive fluorination steps. The alternative methods to direct fluorination with hydrogen fluoride gas are using ammonium difluoride mixed with thorium oxide at room temperature [41] and the thermal degradation of thorium hydrate [42].

Most of the published data on UF_4_ synthesis focus on kinetic studies of the fluorination reaction rate of UO_2_ powder with HF gas [43,44,45,46], rather than on achieving a high-purity product at the gram scale, as in our present work. Since the experimental techniques and setups used in these studies differ from our conditions, a direct comparison of the outcomes is not straightforward. However, our results confirm the high efficiency of the parameters and conditions primarily used in [43,46], with the reaction temperature (450 °C) being particularly important. The studies that focus more on the synthesis of pure UF_4_ are [47,48]. Our findings evidence with the necessity of using a high-surface-area initial oxide, which we prepared by calcining uranium oxalate at 450 °C, as mentioned in [47]. However, we were able to achieve a high-purity product at a lower temperature than the authors of these studies were. A direct comparison of the UF_4_ purities is not possible, as no analytical results are provided in those works.

The method used for synthesizing PuF_3_ in the present work can be compared to similar techniques described in the literature [47,49,50]. In these studies, the conversion of plutonium oxide to tetrafluoride was typically carried out at a similar temperature (550 °C), but the reduction to plutonium trifluoride by hydrogen gas was achieved at significantly lower temperatures, 300 °C, in contrast to the 600 °C used in our experiment [49]. This difference can be attributed to the lower reactivity of hydrogen diluted with argon to 6%, which was used for safety reasons in our laboratory. Several alternative methods for synthesizing PuF_3_ have been published that use different reagents such as nitrogen trifluoride [51], chlorine trifluoride [52], or ammonium difluoride [53]. These methods are often employed to avoid handling both highly radioactive material and highly corrosive gases at high temperatures in the same facility. However, these alternative methods tend to be less effective in producing highly pure material.

### 4.2. Outcomes from the Synthesis of UF_3_

As mentioned in Section 1.3, the method for UF_3_ synthesis was primarily based on the work of Roy [22], but it was adapted to our experimental conditions, allowing the use of only diluted hydrogen gas. As reported in that work, the main difficulties in reducing UF_4_ with H_2_ gas include unfavorable kinetics at lower temperatures, as well as the disproportionation of the UF_3_ product to U metal and UF_4_ at temperatures above 850 °C. The work of Berndt [54] highlighted an additional issue: the tendency of the product to readily react with oxygen and/or moisture in the reduction gas, leading to UF_3_ that is contaminated by UO_2_. The reaction temperatures reported herein differed significantly from those in Roy’s work, as the reduction was performed at 1050 °C, with disproportionation observed only at temperatures above 1080 °C. Additionally, the reaction was not quantitative at temperatures below 950 °C.

Due to the scope of our work, a thorough study on the synthesis, characterization, and parametric optimization of the synthesized materials was not conducted. However, the following basic observations were made:

- The reaction was not quantitative up to a temperature of 800 °C, as some residual UF_4_ was always present at lower temperatures, specifically at 600, 700, and 750 °C;
- Using a flow rate of H_2_/Ar gas lower than 600 mL/min led to disproportionation of the product at 800 °C;
- The reaction kinetics at 800 °C, with a H_2_/Ar gas flow rate of 600 mL/min, were still very slow, requiring 40 h to complete the conversion;
- The commercially available mixture of Ar/H_2_ (6% H_2_, Linde, 99.9999% purity) was insufficient to prevent the formation of trace amounts of UO_2_ in the product (<0.5 wt.%), likely due to oxygen or moisture impurities in this reaction gas.

Our results align well with those reported by Roy, while the reaction and disproportionation temperatures published by Berndt are higher than those observed in our work. On the other hand, we confirmed the risk of contamination by UO_2_ due to oxygen and/or moisture in the reaction gas. We also corroborated the slow reaction kinetics and the need for a long reaction time and a high flow rate of the reduction gas. Although our experimental set-up did not permit the purification of the reduction gas from oxygen and moisture, we achieved fully satisfactory results using a temperature of 800 °C, a H_2_/Ar gas flow rate of 600 mL/min, a reaction time of 40 h, and 1 g of initial UF_4_ material. These process parameters yielded UF_3_ of very high purity, containing only trace amounts of UO_2_ (<0.5 wt.%) and no other phases. The scope of our work did not allow for a detailed study, which we plan to undertake in the future, using a wider range of reaction parameters and employing gas purification techniques.

### 4.3. Outcomes from Production of Solid Melt Ingots

The production of solid melt ingots from mixtures of actinide fluorides was a highly specific and unique task, designed explicitly for the given experimental conditions, as described in detail in Section 3.2. Consequently, no relevant literature was found to compare our results with previously published works. The main outcome from this work was the finding that simply melting the fluoride powders and allowing the melts to cool under a purified Ar atmosphere was insufficient, as the ingots produced in this manner developed an oxide layer on their surface that could not be removed. As discussed in Section 3.2, the source of oxygen was likely the Ar cover gas. However, it was proven that, when the melting and cooling were carried out under an HF cover gas, the ingots had only an extremely thin oxide layer on their surface, which could be easily removed mechanically, yielding perfectly pure ingots.

## 5. Conclusions, Implications, and Future Research

The SALIENT-03 experiment represents the first irradiation study using molten salt containing thorium, uranium, and plutonium since the work conducted in the Oak Ridge National Laboratory in the 1960s–1970s. This experiment holds significant potential to advance the evaluation of safety aspects of the Molten Salt Reactor concept, an innovative nuclear reactor design that can play a crucial role in the decarbonization of global electricity generation. This study focused on the synthesis, purification, and characterization of the molten salt fuel required for the irradiation experiment. The process included the synthesis of necessary fluorides from readily available materials, the mixing and homogenization of different fuel compositions, and the fabrication of salt ingots that are compatible with irradiation capsules.

The key outcomes of this work can be summarized as follows:

- The fuels for the SALIENT-03 irradiation experiment have been successfully synthesized with the required composition, purity, and mass;
- The fuel ingots have been inserted into irradiation capsules, sealed in a gas-tight manner by orbital welding, and transported from JRC Karlsruhe to NRG Petten, where they have been accepted for the irradiation campaign;
- The achieved purity of all end-members synthesized during this work exceeded 99.0%, based on the detection limit and uncertainty of the applied analytical techniques;
- No impurities have been detected in the end-members, except for UF_3_, where trace amounts of oxide impurity (<0.5%) were observed;
- The synthesis campaign detailed herein has demonstrated the capability of JRC laboratories to prepare fluoride salts of high purity, including actinide fluorides, on a 100 g scale, since the total mass of the three fuels created for irradiation is 40.6 g, and the mass of the fuel for the out-of-pile electrochemical experiments is 53.8 g.

With all the goals of this experiment having been successfully achieved, the results offer promising prospects for future research and the development of fuel production methods for fluoride-based molten salt reactors. The synthesized quantities of actinide fluorides have significantly exceeded typical laboratory synthesis scales, while maintaining exceptional purity. Although no specific data regarding acceptable impurity levels for MSR fuel have been found in the literature, it is highly plausible that the achieved purity level of over 99% would be sufficient for the safe and sustainable operation of reactors. This work has also highlighted the complexities involved in synthesizing fuel salts for MSR applications in laboratory conditions. In the future research and development of MSR fuel production, we recommend paying special attention to further scale-up effects as well as quality control and assurance.

Future work at JRC Karlsruhe will include studies on methods for direct oxygen analysis in the synthesized actinide halides and fuel salts. While technically challenging, this analysis is critical for the future qualification of any MSR fuels. Additionally, our research will continue into the synthesis and characterization of alternative MSR fuel candidates, such as actinide chlorides, while we maintain expertise and a unique experimental facility for the production of MSR fuels for potential future irradiation experiments.

## Figures and Tables

**Table 1:** Compositions of salt mixtures for SALIENT-03 and the required masses to be synthesized.

Salt Composition (mol.%)	Mass (g)	Acronym
75.0^7^LiF—18.7ThF_4_—6.0UF_4_—0.3PuF_3_	23.151	Fuel-1
75.0^7^LiF—18.7ThF_4_—5.7UF_4_—0.3UF_3_—0.3PuF_3_	11.584	Fuel-2
74.6^7^LiF—18.6ThF_4_—6.0UF_4_—0.4CrF_3_ -0.3PuF_3_	5.792	Fuel-3
75.0LiF—23.0ThF_4_—2.0UF_4_—0.1UF_3_	50.000	Fuel-4

**Table 2:** Masses of end-members to be synthesized to prepare the complete SALIENT-03 fuel, including 20% extra material allocated for sampling for QA and losses.

End-Member	^7^LiF	ThF_4_	UF_4_	UF_3_	PuF_3_
Mass required (g)	10.075	66.423	12.811	0.282	0.459

**Table 3:** Distribution of the salt mixtures to SALIENT-03 irradiation capsules.

Pin	Material	Salt	Mass (g)
1	Hastelloy N	Fuel-1	11.567
2	Hastelloy N	Fuel-2	5.792
3	Hastelloy N	Fuel-1	5.792
4	Hastelloy N	Fuel-3	5.792
5	GH3535	Fuel-2	5.792
D1	Hastelloy N	Fuel-1	5.792

**Table 4:** Parameters, mass balance, and results of the ThF_4_, UF_4_, and PuF_3_ syntheses (*m*_AnFx_: mass of the product after the reaction, *m*_AnFx_FINAL_: mass of the recovered product after sampling).

Batch	AnO_2_ Mass (g)	T (°C)	Time (h)	*m*_AnFx_ (g)	Conversion Efficiency	XRD	DSC (m.p.°C)	*m*_AnFx_FINAL_ (g)
ThF4-1	15.4910	600	5:15	17.8071	99.1%	phase pure	1117.0	17.5865
ThF4-2	16.0160	600	5:40	18.3997	98.4%	phase pure	1118.6	18.2528
ThF4-3	15.5905	600	5:45	17.2990	n/a ^1^	phase pure	1119.4 ^2^	17.1193
ThF4-4	15.8677	600	5:15	18.5115	98.5%	phase pure	1119.4 ^2^	17.3325
UF4-1 ^3^	7.5841	450	6:30	8.7385	99.1%	phase pure	1029.5	8.5123
UF4-2 ^3^	9.6630	450	7:00	11.1562	99.3%	phase pure	1016.5	10.7598
PuF_3_^4^	2.8163	550	2:10	n/a ^5^	n/a ^5^	n/a ^5^	n/a ^5^	n/a ^5^
		600	15:00	3.0708	99.8%	phase pure	n/a ^6^	2.9239

^1^ Not available (n/a), because 0.4583 g of partly fluorinated initial material was lost from the boat during the reaction. ^2^ Analyzed in a mixture of all four batches. ^3^ UF_4_ synthesis was carried out in two steps without analyzing the intermediate product. The conversion efficiency is given for the product after the first fluorination step, as the manipulation losses between the first and second step do not allow for evaluation of the overall efficiency. ^4^ The first line: fluorination, second line: reduction. ^5^ Not available (n/a), because the PuF_3_ synthesis was carried out without analyzing the intermediate product. ^6^ Not available (n/a), because melting point of PuF_3_ is higher than the encapsulating material used for the DSC measurement.

**Table 5:** Mass balance and results of the UF_3_ synthesis.

Batch	UF_4_ Mass (g)	T (°C)	Time (h)	UF_3_ Mass (g)	Conversion Efficiency	XRD	DSC (m.p.°C)	Final Mass (g)
UF3 03/19	1.0807	800	40:00	0.9797	96.5% ^1^	>99.5% UF_3_<0.5% UO_2_	n/a ^2^	0.7841

^1^ Sublimation/evaporation of the product indicated by condensed deposits in the reactor; however, they are technically impossible to recover and quantify. ^2^ UF_3_ disproportionation before reaching the melting temperature.

**Table 6:** Mass balance of mixing and homogenizing of the fuel salts (*m*_FUEL_: the total mass of all added end-members, *m*_FINAL_: final mass after homogenizing).

Fuel	^7^LiF (g)	ThF_4_ (g)	UF_4_ (g)	UF_3_ (g)	PuF_3_ (g)	CrF_3_ (g)	*m*_FUEL_ (g)	*m*_FINAL_ (g)
1	5.6451	16.6662	5.4535	-	0.2571	-	27.7669	27.7135
2	3.0270	8.9347	2.7844	0.1308	0.1378	-	15.0148	14.9957
3	1.4030	4.1426	1.3613	-	0.0644	0.0351	7.0048	6.9930
4	11.0803 ^1^	40.3503	3.5446	0.0333	-	-	55.0085	54.9448

^1^ not enriched LiF.

**Table 7:** List of all fabricated sub-ingots (*m*_FINAL_: mass of the sub-ingot after melting, Δ*m*_MELTING_: mass difference in the powder salt before melting and the sub-ingot, *m*_BLACK_L_: estimated mass of the black layer on the sub-ingot surface, n/a—not available due to losses during manipulation).

ID	*m*_FINAL_ (g)	Δ*m*_MELTING_ (g/%)	*m*_BLACK_L_ (g)	Target Pin	Composition (mol.%)
1-1	2.9324	0.0086/0.29	0.0171	1	75.0^7^LiF—18.7ThF_4_ 6.0UF_4_—0.3PuF_3_
1-2	2.9806	0.0094/0.31	0.0095		
1-3	2.9599	0.0088/0.30	0.0103		
1-4	2.7222	0.0079/0.29	0.0059		
2-1	1.6925	n/a	0.0053	2	75.0^7^LiF—18.7ThF_4_—5.7UF_4_—0.3UF_3_—0.3PuF_3_
2-2	1.3953	0.0035/0.25	0.0061		
2-3	1.3455	0.0027/0.20	0.0024		
2-4	1.3891	0.0032/0.23	0.0022		
3-1	1.8507	0.0030/0.16	0.0079	3	75.0^7^LiF—18.7ThF_4_ 6.0UF_4_—0.3PuF_3_
3-2	1.3526	0.0039/0.29	0.0028		
3-3	1.3227	n/a	0.0023		
3-4	1.2800	0.0036/0.28	0.0020		
4-1	1.4837	0.0044/0.30	0.0020	4	74.6^7^LiF—18.6ThF_4_—6.0UF_4_—0.4CrF_3_—0.3PuF_3_
4-2	1.5049	0.0039/0.26	0.0033		
4-3	1.4736	0.0043/0.29	0.0023		
4-4	1.3569	0.0039/0.29	0.0033		
5-1	1.5086	0.0034/0.22	0.0034	5	75.0^7^LiF—18.7ThF_4_—5.7UF_4_—0.3UF_3_—0.3PuF_3_
5-2	1.3070	n/a	0.0017		
5-3	1.1841	n/a	0.0020		
D-1	1.6061	0.0063/0.39	0.0057	D	75.0^7^LiF—18.7ThF_4_ 6.0UF_4_—0.3PuF_3_
D-2	1.5822	0.0016/0.10	0.0004		
D-3	1.4849	0.0043/0.29	0.0030		
D-4	1.1511	0.0031/0.27	0.0017		
GC-1	0.4271	0.0012/0.28	0.0014	5	75.0^7^LiF—18.7ThF_4_—5.7UF_4_—0.3UF_3_—0.3PuF_3_
GC-2	0.3974	0.0003/0.08	0.0001		
GC-3	0.4153	0.0015/0.36	0.0010		
GC-4	0.4014	n/a	0.0009		
GC-5	0.4365	0.0010/0.23	0.0004	QA	
GC-6	0.4093	0.0009/0.22	0.0010		

**Table 8:** Mass balance of the sub-ingots from Fuel-1 for capsule 1 (*m*_SALT_: mass of the powder before melting, *m*_INGOT_: mass of the sub-ingot before melting, ∆*m*_MELTING_: mass loss during melting, *m*_FINAL_: mass of the sub-ingot after melting, *m*_BLACK_L_: estimated mass of the black layer on the sub-ingot surface, ∆*m*_LINER_: mass difference of the liners before and after melting).

Capsule 1	Sub-Ingot 1-1	Sub-Ingot 1-2	Sub-Ingot 1-3	Sub-Ingot 1-4
*m*_SALT_ (g)	2.9581	2.9995	2.9790	2.7360
*m*_INGOT_ (g)	2.9495	2.9901	2.9702	2.7281
Δ*m*_MELTING_ (g/%)	0.0086/0.29	0.0094/0.31	0.0088/0.30	0.0079/0.29
*m*_FINAL_ (g)	2.9324	2.9806	2.9599	2.7222
*m*_BLACK_L_ (g/%)	0.0171/0.58	0.0095/0.32	0.0103/0.35	0.0059/0.22
Δ*m*_LINER_ (g/%)	0.0002/0.002	0.0002/0.001	0.0002/0.002	0.0002/0.002
Photo	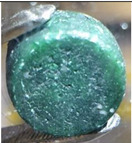	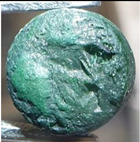	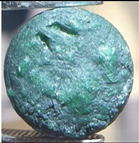	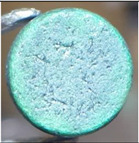

**Table 9:** Mass balance of the final ingots (*m*_FINAL_: mass of the final ingot after melting, Δ*m*_MELTING_: mass difference of the powder salt before melting and the prepared sub-ingot, *m*_BLACK_L_: estimated mass of the black layer on the final ingot surface, Δ*m*_REQUEST_: difference between the ingot mass and the requested mass, density: density of the ingots estimated from basic geometrical determination of the volume and *m*_FINAL_).

Target Capsule	*m*_FINAL_ (g)	Δ*m*_MELTING_ (g/%)	*m*_BLACK_L_ (g)	Δ*m*_REQUEST_ (%)	Density (g/cm^3^)
1	11.5850	0.0021/0.02	0.0119	0.16	4.85
2	5.8039	0.0011/0.02	0.0076	0.21	4.40
3	5.7987	0.0017/0.03	0.0119	0.12	4.49
4	5.8182	0.0029/0.05	0.0030	0.45	4.55
5	5.7947 ^1^	0.0022/0.03^1^	0.0031 ^1^	0.05	4.30
D1	5.8047	0.0010/0.02	0.0037	0.22	4.35

^1^ Sum of masses of the ingots added directly to the pin and the ingots prepared in the cylindrical liner.

## Data Availability

The original contributions presented in the study are included in the article/Supplementary Material, further inquiries can be directed to the corresponding authors.

## Supplementary Materials

The following supporting information can be downloaded at: https://www.mdpi.com/article/10.3390/ma17246215/s1, Figure S1. XRD pattern of the synthesized ⁷LiF. All identified peaks correspond to the LiF phase (ICSD database reference code 98-001-8012). Rietveld refinement could not be performed due to a very high background, likely caused by the presence of a low-crystalline or amorphous phase. However, DSC analysis confirmed the excellent chemical purity of the product, and the potential presence of such a phase was therefore considered negligible. Figure S2. Melting point determination of the synthesized ^7^LiF; the determined melting temperature after calibration correction is 846.8 °C (the corrected value differs from the graph). Figure S3 Rietveld refinement of the XRD pattern obtained from the ThF_4_ synthesis product. The crystallographic cell parameters for the single detected phase ThF_4_, determined from the Rietveld refinement, are shown in the graph. Figure S4. Melting point determination of the synthesized ThF_4_; the determined melting temperature after calibration correction is 1118.6 °C (the corrected value differs from the graph). Figure S5. Rietveld refinement of the XRD pattern obtained from the UF_4_ synthesis product. The crystallographic cell parameters for the single detected phase UF_4_, determined from the Rietveld refinement, are shown in the graph. Figure S6. Melting point determination of the synthesized UF_4_; the determined melting temperature after calibration correction is 1029.5 °C (the corrected value differs from the graph). Figure S7. Rietveld refinement of the XRD pattern obtained from the PuF_3_ synthesis product. The crystallographic cell parameters for the single detected phase PuF_3_, determined from the Rietveld refinement, are shown in the graph. Figure S8. Melting point determination of the synthesized Fuel-1 mixture; the determined melting temperature after calibration correction is 547.6 °C (the corrected value differs from the graph). Figure S9 Melting point determination of the synthesized Fuel-2 mixture; the determined melting temperature after calibration correction is 549.2 °C (the corrected value differs from the graph). Figure S10. Melting point determination of the synthesized Fuel-3 mixture; the determined melting temperature after calibration correction is 549.2 °C (the corrected value differs from the graph). Table S1 Association between the XRD and DSC results of the analysed materials and the figures. Table S2. Procedure for the synthesis of ^7^LiF. Table S3. Procedure for the synthesis of ThF_4_. Table S4. Procedure for the synthesis of UF_4_. Table S5. Procedure for the synthesis of PuF_3_. Table S6. Procedure for the synthesis of UF_3_. Table S7. Procedure for mixing and homogenizing the fuel salts.
