# Supplementary material for: Synthesis, Purification, and Characterization of Molten Salt Fuel for the SALIENT-03 Irradiation Experiment
Source: Materials (Basel). 2024 Dec 19;17(24):6215. doi: 10.3390/ma17246215 (PMC11678894; doi:10.3390/ma17246215)
Supplement: Supplementary file 1 [file materials-17-06215-s001.zip › materials-3330885-supplementary.pdf]

Supporting Material: Annex S1

Analyses of the end-members and fuel mixtures

Annex 1 summarizes the representative results of the X-ray diffraction (XRD) and differential scanning calorimetry (DSC) of the end-members and the molten salt fuel synthesized for the irradiation experiment SALIENT-03. The analyses of UF_3 and CrF_3 are not included in this Annex, as the results for UF_3 are included in this article and the analysis of CrF_3 was omitted as justified in detail in Section 3.2 in this article.

The XRD samples were prepared by embedding approximately 20–25 mg of the product into a resin, while the powder was homogenized beforehand by manual grinding in an agate mortar. The XRD measurements were carried out using a Bruker D8 Bragg-Brentano Advance diffractometer (Cu Kα1 radiation) equipped with a LynxEye Linear Position Sensitive detector (Bruker AXS GmbH, Karlsruhe, Germany). The operation conditions were 40 kV and 40 mA. Powder diffraction patterns were recorded at room temperature, typically across an angular range of $10° \leq 2\theta \leq 120°$. The phase quantification procedure involved in the identification of the different phases was achieved using Jana 2006 crystallographic software (Institute of Physics, Czech Academy of Sciences, Prague, Czech Republic).

The DSC measurements were carried out with a Setaram multi detector high temperature MDHTC-96 type calorimeter (Setaram Instrumentation, Caluire-et-Cuire, France) with a DSC detector based on S-type thermocouples with a peak temperature of 1400 °C. The program for melting temperature determination consisted of three heating and cooling ramps at a rate 10 K/min. To avoid the evaporation of fluoride samples, both materials were encapsulated in gas-tight, high pressure resistant stainless steel crucibles with inserted nickel liners to prevent any undesired reaction between fluoride salt and the steel, as detailed in [28]. The calorimeter was calibrated by a series of standard materials (In, Sn, Zn, Pb, Al, Ag, Au) of different melting points, placed in the same crucibles to correct for the offset temperature during the measurement. The uncertainty of the measurements was determined as ±5 °C.

Table S1 presents the association between the XRD and DSC results of the analyzed materials and the figures provided below.

Table S1. Association between the XRD and DSC results of the analyzed materials and the figures.

**Method**	**Analyzed material**							
	**^{7}LiF**	**ThF_4**	**UF_4**	**UF_3**	**PuF_3**	**Fuel-1**	**Fuel-2**	**Fuel-3**
XRD	Figure S1	Figure S3	Figure S5	Figure 2*	Figure S7	n/a	Figure 5*	n/a
DSC	Figure S2	Figure S4	Figure S6	n/a	n/a	Figure S8	Figure S9	Figure S10

* In the main text of the article

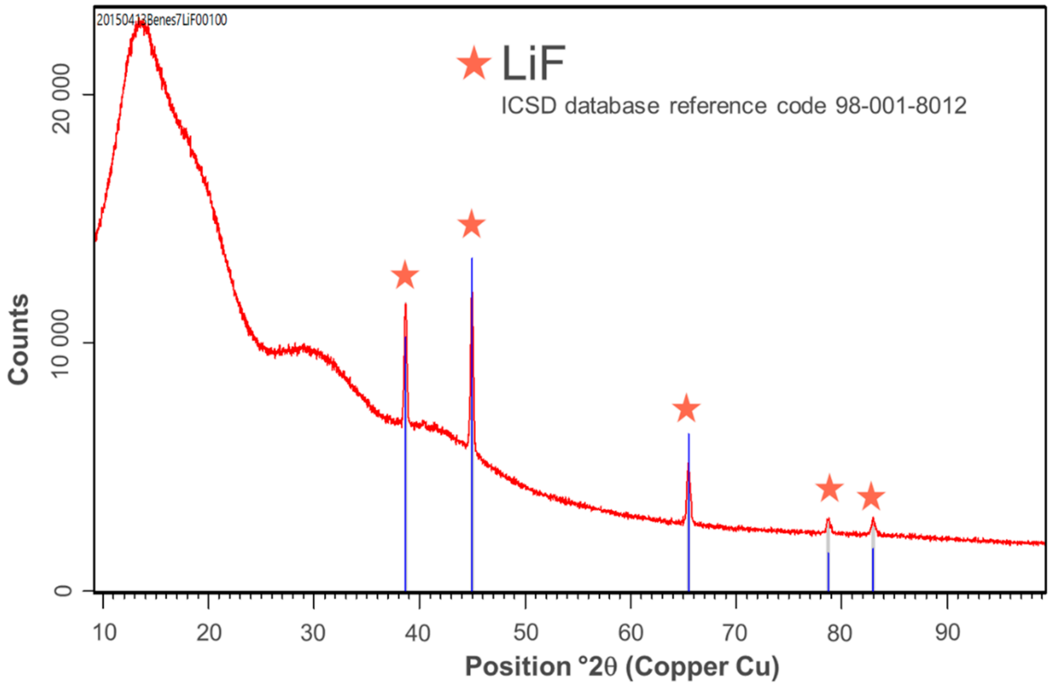

Figure S1. XRD pattern of the synthesized [7]LiF. All identified peaks correspond to the LiF phase (ICSD database reference code 98-001-8012). Rietveld refinement could not be performed due to a very high background, likely caused by the presence of a low-crystalline or amorphous phase. However, DSC analysis confirmed the excellent chemical purity of the product, and the potential presence of such a phase was therefore considered negligible.

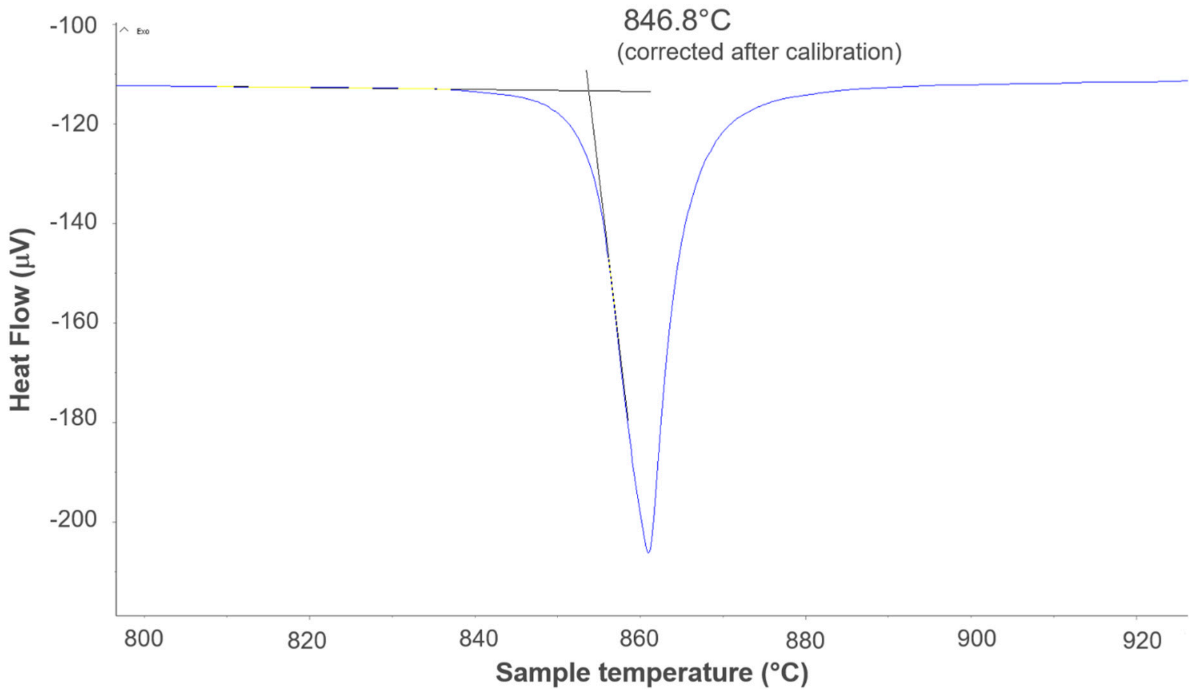

Figure S2. Melting point determination of the synthesized [7]LiF; the determined melting temperature after calibration correction is 846.8 °C (the corrected value differs from the graph).

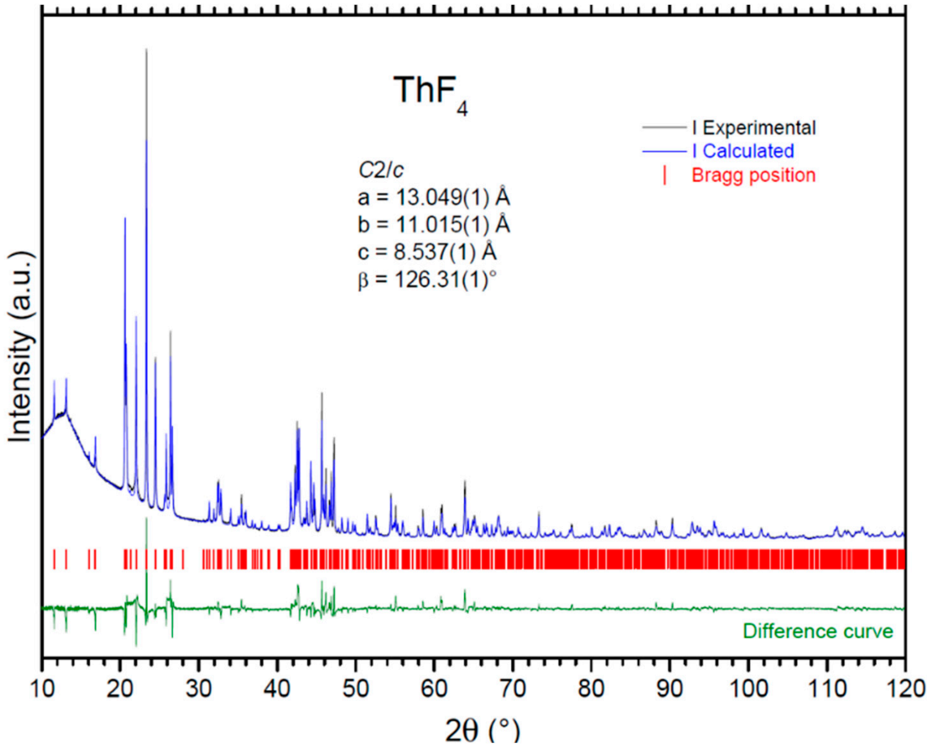

Figure S3. Rietveld refinement of the XRD pattern obtained from the ThF_4 synthesis product. The crystallographic cell parameters for the single detected phase ThF_4, determined from the Rietveld refinement, are shown in the graph.

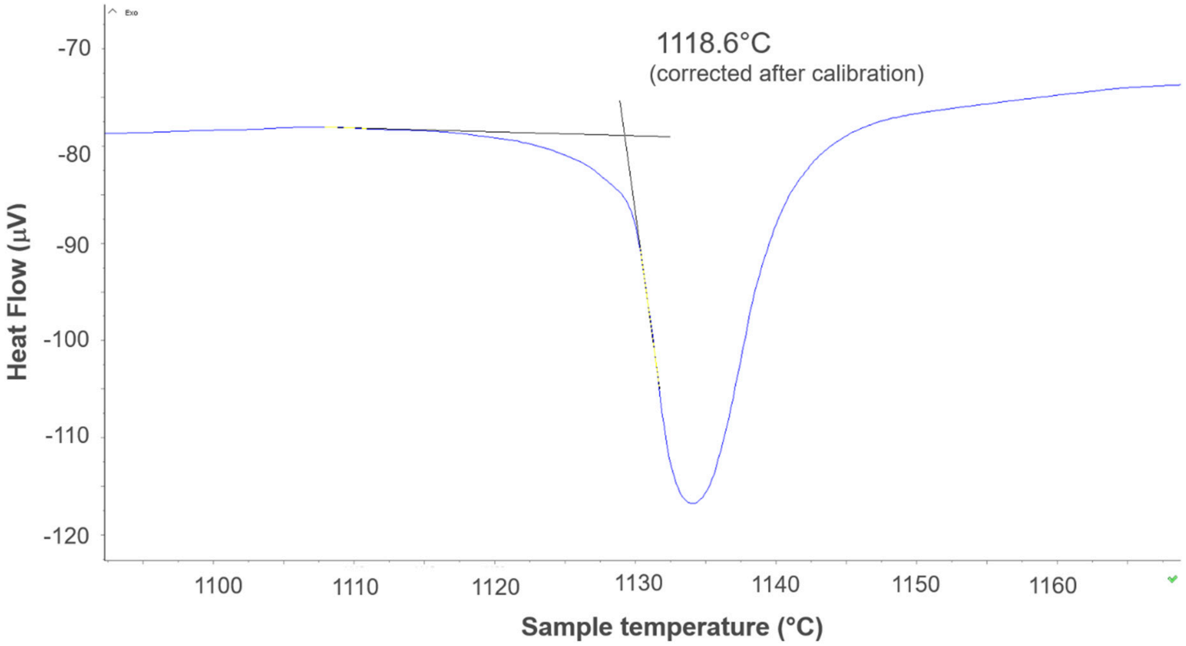

Figure S4. Melting point determination of the synthesized ThF_4; the determined melting temperature after calibration correction is 1118.6 °C (the corrected value differs from the graph).

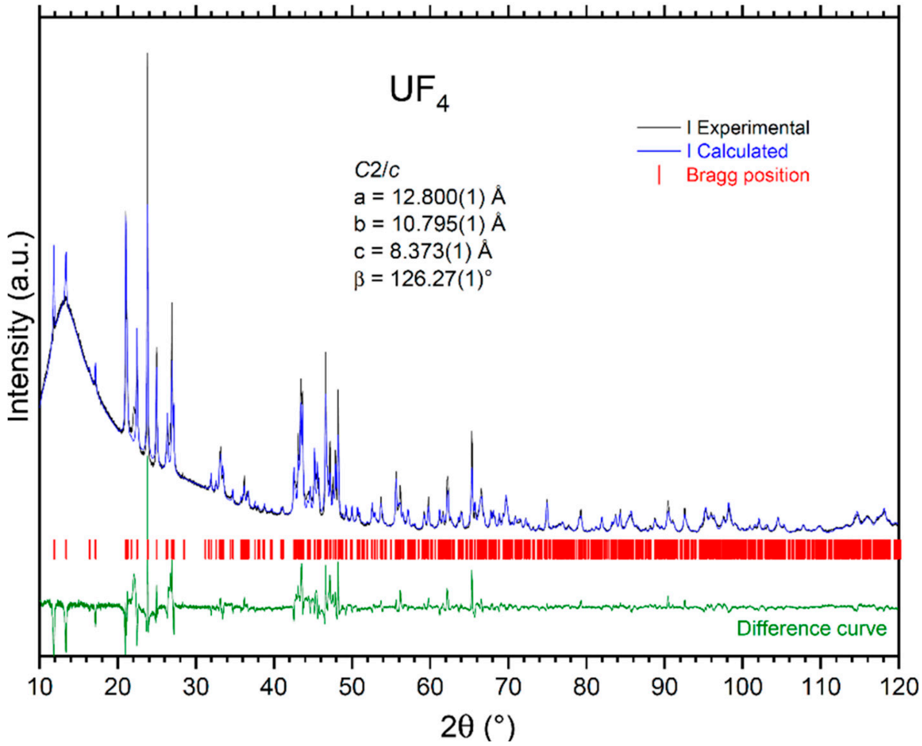

Figure S5. Rietveld refinement of the XRD pattern obtained from the UF_4 synthesis product. The crystallographic cell parameters for the single detected phase UF_4, determined from the Rietveld refinement, are shown in the graph.

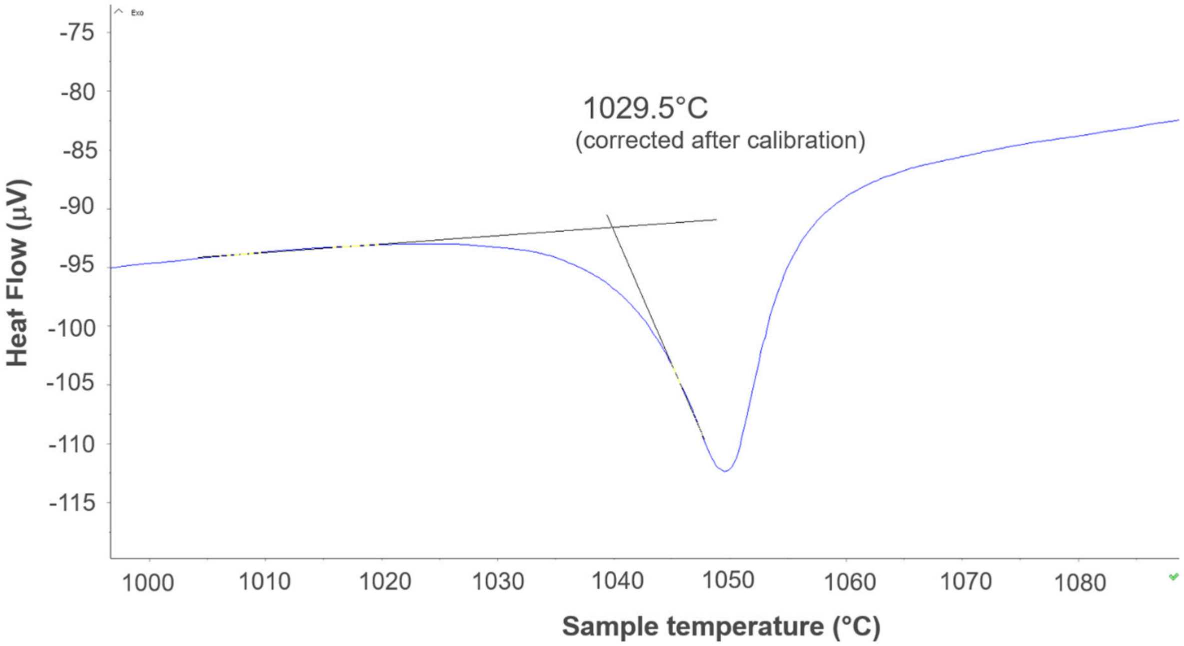

Figure S6. Melting point determination of the synthesized UF_4; the determined melting temperature after calibration correction is 1029.5 °C (the corrected value differs from the graph).

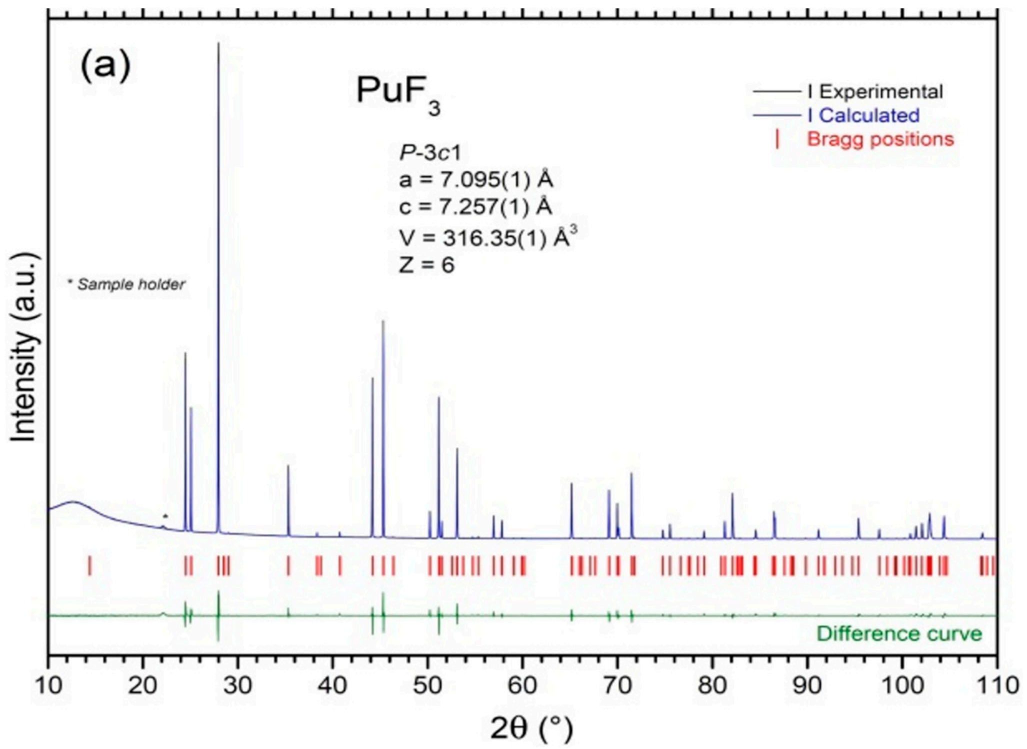

Figure S7. Rietveld refinement of the XRD pattern obtained from the PuF_3 synthesis product. The crystallographic cell parameters for the single detected phase PuF_3, determined from the Rietveld refinement, are shown in the graph.

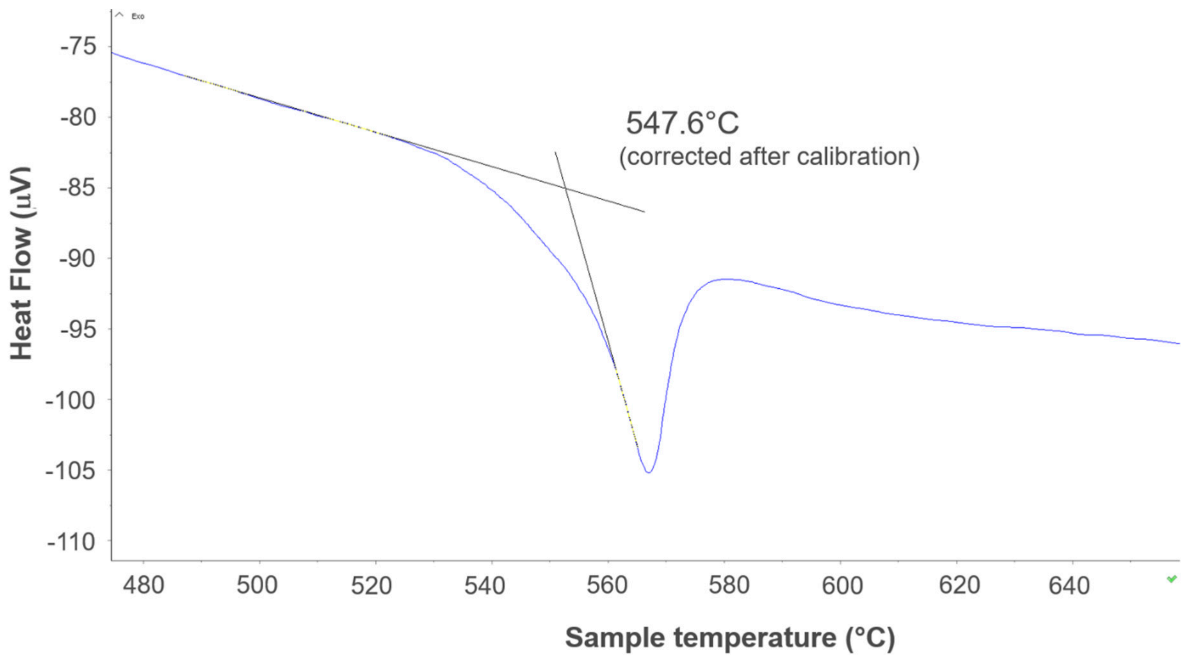

Figure S8. Melting point determination of the synthesized Fuel 1 mixture; the determined melting temperature after calibration correction is 547.6 °C (the corrected value differs from the graph).

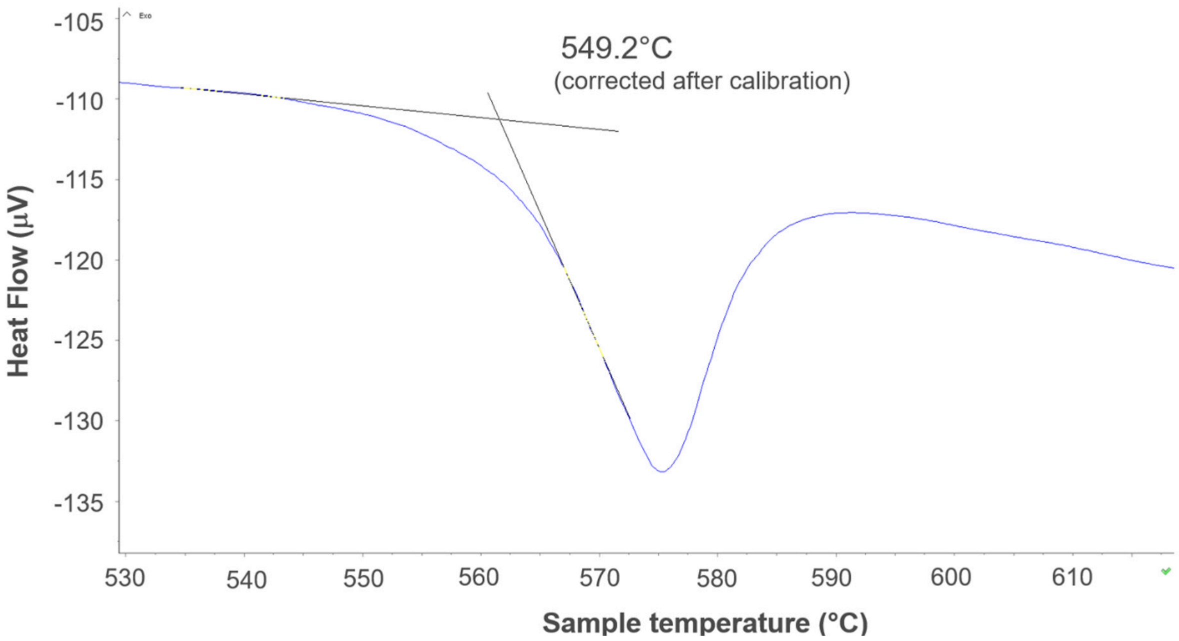

Figure S9. Melting point determination of the synthesized Fuel 2 mixture; the determined melting temperature after calibration correction is 549.2 °C (the corrected value differs from the graph).

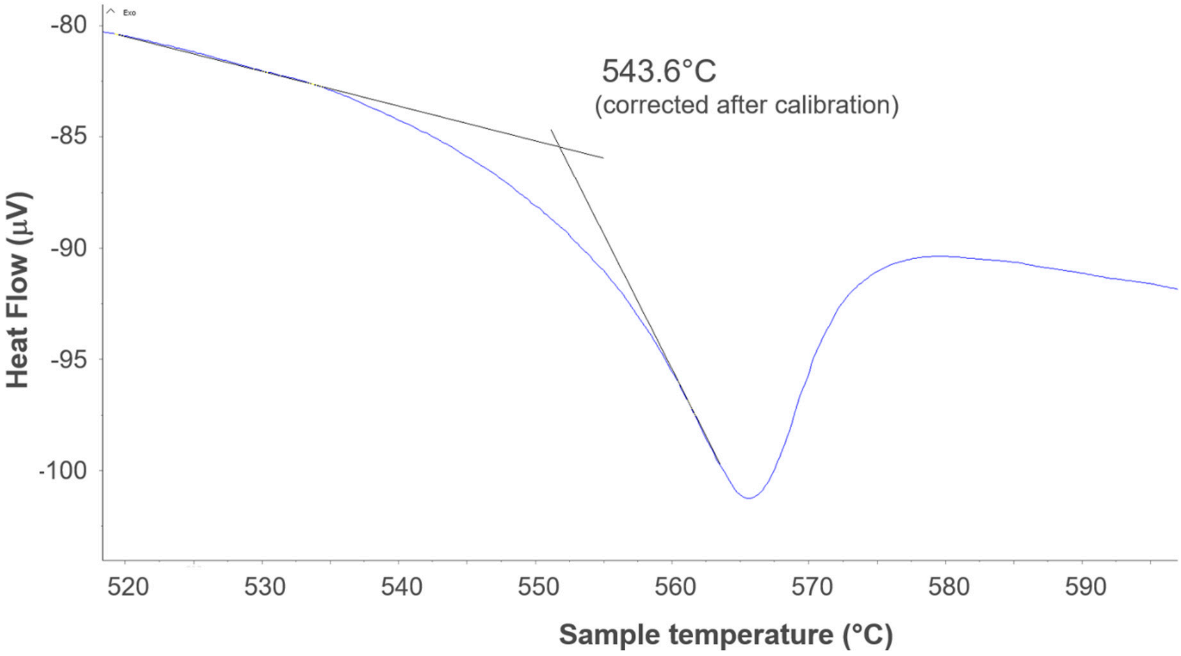

Figure S10. Melting point determination of the synthesized Fuel 3 mixture; the determined melting temperature after calibration correction is 549.2 °C (the corrected value differs from the graph).

Supporting Material: Annex S2

Procedures Used for the Main Steps of the SALIENT-03 Fuel Fabrication

Annex 1 outlines the procedures used to achieve satisfactory results for all the main steps of the SALIENT-03 fuel fabrication process in tabular form. These steps include the synthesis of the end-members ^7LiF, ThF_4, UF_4, UF_3, and PuF_3; the mixing and homogenizing of the required end-members to form the fuels; and the preparation of the fuel ingots. A brief summary of the influence of parameter changes on the results, when available, is provided below each table. However, parametric studies on the process parameters were in principle not within the scope of the present work.

Table S2. Procedure for the synthesis of ^7LiF

Process step	Reactants	Process conditions
Dissolution	$^7LiOH{\cdot}H_2O$	Dissolution in water to concentration 2 M, room temperature
Filtration	$^7Li_2CO_3$ contained in the package of $^7LiOH{\cdot}H_2O$	Standard filtration in a Büchner funnel at room temperature, vacuum ~50 mBar
Conversion	Solutions of $^7LiOH{\cdot}H_2O$ and 2 M HF acid	Online controlled by a pH meter with a calibrated pH electrode to pH ~4-5
Filtration	Formed ^7LiF	Standard filtration in a Büchner funnel at room temperature, vacuum ~50 mBar
Purification	Obtained filter cake ^7LiF	Rinsing with pure ethanol
Drying 1. step	^7LiF product	Laboratory dryer, air, 150°C, 2h
Drying 2. step	^7LiF product	Furnace in a glove box, argon gas, 350°C, 2 h

If filtration of $^7Li_2CO_3$ is not done, the process efficiency decreases significantly, based on the mass of the $^7Li_2CO_3$ contained in the original package of $^7LiOH{\cdot}H_2O$, and drying under air is not sufficient to achieve a sufficiently dehydrated product.

Table S3. Procedure for the synthesis of ThF_4.

Process step	Reactants	Process conditions
Preparation	ThO_2	Powder filled in a boron nitride boat and inserted in reactor
Heating	ThO_2, Ar gas	Temperature 600°C, ramp 10°C/min, Ar flow rate 100 mL/min
Conversion	ThO_2, HF gas	Flow through, HF flow rate 50 mL/min, min. 5 h
Cooling	ThF_4, Ar gas	Room temperature, ramp 10°C/min, Ar flow rate 100 mL/min
Processing	ThF_4	Crushing in a mortar under Ar, sampling

Temperatures lower than 600 °C result in incomplete conversion, with the product containing traces of oxide. Higher temperatures were not tested, as the kinetics at the optimized temperature were sufficient for the desired outcome.

Table S4. Procedure for the synthesis of UF_4

Process step	Reactants	Process conditions
Preparation	UO_2	Powder filled in a boron nitride boat and inserted in reactor
Heating	UO_2, Ar gas	Temperature 450°C, ramp 10°C/min, Ar flow rate 100 mL/min
Conversion step 1	UO_2, HF gas	Flow through, HF flow rate 50 mL/min, min. 4 h
Cooling	UF_4, residual UO_2, Ar gas	Room temperature, ramp 10°C/min, Ar flow rate 100 mL/min
Processing	UF_4, residual UO_2	Crushing in a mortar under Ar, sampling
Heating	UF_4, residual UO_2, Ar gas	Temperature 450°C, ramp 10°C/min, Ar flow rate 100 mL/min
Conversion step 2	UO_2, residual UO_2, HF gas	Flow through, HF flow rate 50 mL/min, min. 2 h
Cooling	UF_4, Ar gas	Room temperature, ramp 10°C/min, Ar flow rate 100 mL/min
Processing	UF_4	Crushing in a mortar under Ar, sampling

Temperatures below 450 °C result in incomplete conversion, with the product containing traces of oxide. Higher temperatures were not tested, as thermodynamic modelling indicated the potential reconversion of UF_4 to UO_2 at elevated temperatures. Two conversion steps, with crushing of the intermediate product between steps, were found to be necessary, even when the reaction time in the first step was extended to 6 h.

Table S5. Procedure for the synthesis of PuF_3

Process step	Reactants	Process conditions
Preparation	PuO_2	Powder filled in a boron nitride boat and inserted in reactor
Heating	PuO_2, Ar gas	Temperature 550°C, ramp 10°C/min, Ar flow rate 100 mL/min
Conversion	PuO_2, HF gas	Flow through, HF flow rate 50 mL/min, min. 2 h
Heating	PuF_4, H_2/Ar (6%H_2) gas	Temperature 600°C, ramp 5°C/min, H_2/Ar (6%H_2) flow rate 100 mL/min
Reduction	PuF_4, H_2/Ar (6%H_2) gas	Flow through, H_2/Ar flow rate 100 mL/min, min. 15 h
Cooling	PuF_3, Ar gas	Room temperature, ramp 10°C/min, Ar flow rate 100 mL/min
Processing	PuF_3	Crushing in a mortar under Ar, sampling

Temperatures below 550 °C and 600 °C result in incomplete conversion and reduction, respectively. Higher temperatures were not tested due to time and material availability constraints.

Table S6. Procedure for the synthesis of UF_3

Process step	Reactants	Process conditions
Preparation	UF_4	Powder filled in a nickel boat and inserted in reactor
Heating	UF_4, Ar gas	Temperature 800°C, ramp 10°C/min, Ar flow rate 100 mL/min
Reduction	UF_4, H_2/Ar (6%H_2) gas	Flow through, H_2/Ar flow rate 600 mL/min, min. 40 h
Cooling	UF_3, H_2/Ar (6%H_2) gas	Room temperature, ramp 10°C/min, H_2/Ar (6%H_2) flow rate 100 mL/min
Processing	UF_3	Crushing in a mortar under Ar, sampling

Temperatures below 800 °C result in incomplete conversion, with the product containing traces of UF_4. Higher temperatures and, in the same amount of time, a lower H_2/Ar gas flow rate lead to disproportionation of the product to UF_4 and U metal.

Table S7. Procedure for mixing and homogenizing the fuel salts

Process step	Reactants	Process conditions
Mixing	Required end-members	Step-by-step addition of each member to a mixing bottle (min. 2 x volume then sum of all powders)
Mixing step 1	Required end-members	Mixing by a spoon in the bottle, min. 10 min
Mixing step 1	Required end-members	Shaking the bottle, manually, min. 30 min
Homogenising	Required end-members	Grinding in a large agate mortar

Only mixing by a spoon and shaking the bottle is not sufficient to homogenize the mixture, as proven by DSC measurements.

Table S8. Procedure for preparation of the fuel ingots

Process step	Reactants	Process conditions
Preparation	Mixed fuel salt powder	Filling of powders into glassy carbon crucibles, mechanical compaction, inserting into reactor
Heating step 1	Fuel salt powder, Ar gas	Temperature 200°C, ramp 10°C/min, HF flow rate 50 mL/min
Heating step 2	Fuel salt powder, HF gas	Temperature 700°C, ramp 10°C/min, HF flow rate 50 mL/min
Melting	Fuel salt powder, HF gas	Temperature 700°C, 30 min, HF flow rate 50 mL/min
Cooling step 1	Fuel salt powder, HF gas	Temperature 300°C, fast cooling, HF flow rate 50 mL/min
Cooling step 2	Fuel salt ingot, HF gas	Temperature 100°C, fast cooling, HF in closed reactor (flow stopped)
Cooling step 3	Fuel salt ingot, Ar gas	Room temperature, fast cooling, Ar flow rate 100 mL/min
Cleaning	Fuel salt ingot	Mechanical cleaning by brush, rinsing in ethanol, drying by tissue paper, weighting

Melting of the fuel salt powders under an available argon atmosphere containing approximately 2–5 ppm of O_2 is not sufficient as the ingots prepared in this way are covered with an oxide layer, which could not be mechanically removed. Melting using HF cover gas was found necessary. The heating and cooling procedures were not optimized due to time constraints and can be potentially simplified.
